# Imputation of microsatellite alleles from dense SNP genotypes for parentage verification across multiple *Bos taurus* and *Bos indicus* breeds

**DOI:** 10.3389/fgene.2013.00176

**Published:** 2013-09-18

**Authors:** Matthew C. McClure, Tad S. Sonstegard, George R. Wiggans, Alison L. Van Eenennaam, Kristina L. Weber, Cecilia T. Penedo, Donagh P. Berry, John Flynn, Jose F. Garcia, Adriana S. Carmo, Luciana C. A. Regitano, Milla Albuquerque, Marcos V. G. B. Silva, Marco A. Machado, Mike Coffey, Kirsty Moore, Marie-Yvonne Boscher, Lucie Genestout, Raffaele Mazza, Jeremy F. Taylor, Robert D. Schnabel, Barry Simpson, Elisa Marques, John C. McEwan, Andrew Cromie, Luiz L. Coutinho, Larry A. Kuehn, John W. Keele, Emily K. Piper, Jim Cook, Robert Williams, Bovine HapMap Consortium, Curtis P. Van Tassell

**Affiliations:** ^1^Bovine Functional Genomics Laboratory, BARC, Agriculture Research Service, United States Department of AgricultureBeltsville, MD, USA; ^2^Animal Improvement Programs Laboratory, BARC, Agriculture Research Service, United States Department of AgricultureBeltsville, MD, USA; ^3^Department of Animal Science, University of California-DavisDavis, CA, USA; ^4^Veterinary Genetics Laboratory, School of Veterinary Medicine, University of California-DavisDavis, CA, USA; ^5^Animal and Bioscience Research Department, Animal and Grassland Research and Innovation CentreTeagasc, Moorepark, Ireland; ^6^Weatherbys DNA LaboratoryKildare, Ireland; ^7^Sao Paulo State University/UNESPAracatuba, Brazil; ^8^Deoxi BiotecnologiaAraçatuba, Brazil; ^9^Embrapa Pecuária SudesteSão Carlos, Brazil; ^10^Department of Animal Science, College of Agriculture, University of Sao PauloPiracicaba, Brazil; ^11^Embrapa Gado de LeiteJuiz de Fora, Brazil; ^12^Animal and Veterinary Science, SRUCMidlothian, Scotland; ^13^LABOGENAJouy-en-Josas, France; ^14^Coordinatore Laboratorio Genetica e Servizi, Associazione Italiana Allevatori - Italian Breeders AssociationRoma, Italy; ^15^Division of Animal Science, University of Missouri-ColumbiaColumbia, MO, USA; ^16^GeneSeek, Neogen CompanyLincoln, NE, USA; ^17^AgResearch, Invermay Agricultural CentreMosgiel, New Zealand; ^18^Irish Cattle Breeding FederationBandon, Ireland; ^19^ESALQ – USPPiracicaba, SP, Brazil; ^20^Genetics, Breeding, and Animal Health Research Unit, U.S. Meat Animal Research Center, USDA-ARSClay Center, NE, USA; ^21^Animal Genetics Laboratory, School of Veterinary Science, The University of QueenslandGatton, QLD, Australia; ^22^Animal Genetics and Breeding Unit, University of New EnglandArmidale, NSW, Australia; ^23^American-International Charolais AssociationKansas City, MO, USA

**Keywords:** microsatellite, STR, SNP, imputation, parentage verification

## Abstract

To assist cattle producers transition from microsatellite (MS) to single nucleotide polymorphism (SNP) genotyping for parental verification we previously devised an effective and inexpensive method to impute MS alleles from SNP haplotypes. While the reported method was verified with only a limited data set (*N* = 479) from Brown Swiss, Guernsey, Holstein, and Jersey cattle, some of the MS-SNP haplotype associations were concordant across these phylogenetically diverse breeds. This implied that some haplotypes predate modern breed formation and remain in strong linkage disequilibrium. To expand the utility of MS allele imputation across breeds, MS and SNP data from more than 8000 animals representing 39 breeds (*Bos taurus* and B. *indicus*) were used to predict 9410 SNP haplotypes, incorporating an average of 73 SNPs per haplotype, for which alleles from 12 MS markers could be accurately be imputed. Approximately 25% of the MS-SNP haplotypes were present in multiple breeds (*N* = 2 to 36 breeds). These shared haplotypes allowed for MS imputation in breeds that were not represented in the reference population with only a small increase in Mendelian inheritance inconsistancies. Our reported reference haplotypes can be used for any cattle breed and the reported methods can be applied to any species to aid the transition from MS to SNP genetic markers. While ~91% of the animals with imputed alleles for 12 MS markers had ≤1 Mendelian inheritance conflicts with their parents' reported MS genotypes, this figure was 96% for our reference animals, indicating potential errors in the reported MS genotypes. The workflow we suggest autocorrects for genotyping errors and rare haplotypes, by MS genotyping animals whose imputed MS alleles fail parentage verification, and then incorporating those animals into the reference dataset.

## Introduction

Single nucleotide polymorphism (SNP) are preferred to microsatellite (MS) markers for parentage verification and genomic selection due to their higher genotyping accuracies, speed of genotyping, lower overall cost per genotype, and ease of automation. While SNP genotypes per animal (*N* = 3000 to > 7,70,000) assayed on Illumina platforms are routinely > 99% for call rate and concordance (McClure et al., 2009; Rincon et al., 2011), individual MS are known to have a 1–5% genotyping error rate (Baruch and Weller, 2008). When individual genetic markers each have an error rate of 1%, the probability of having at least 1 genotype error in an individual genotyped for 11 MS markers is >10% (Weller et al., 2006). Also, we have observed that single nucleotide insertions or deletions within the amplified MS region can result in the rounding up or down of the called MS allele fragment size resulting in a 2 bp difference in the reported allele size. Therefore, the high inherent chance of genotyping errors has led several studies to suggest that 2 MS marker conflicts must exist for an animal to be excluded in parentage verification (Bonin et al., 2004; Weller et al., 2004; Baruch and Weller, 2008). In a comparison of a bovine parentage MS panel vs. a 32 SNP parentage panel (Heaton et al., 2002) employed for sire discovery for 287 calves from US beef and dairy farms, the SNP panel routinely outperformed the MS panel with the SNP panel assigning a sire at 100% probability 81.9% of the time vs. 38.3% of the time for the MS panel (Stewart Bauck, GeneSeek a Neogen Company, Pers. Commun. 3/10/2013). Recent work by Fernández et al. (2013) showed that even in a Brazilian inbred Angus herd that only 24 SNP were needed to obtain the equivalent matching probability (MP) for parental verification as 18 microsatellites. Similarly, 43 SNP provided 2–4 orders of magnitude grater MP than 11 MS in 6 Northern Ireland cattle breeds (Aberdeen Angus, Belgian Blue, Charolais, Holstein, Limousin, and Simmental) (Allen et al., 2010).

SNP technology is not only used in numerically large breeds, such as Holstein and Angus, but also by numerically mid-size and small breeds for the identification of genetic disease carriers and for genomic selection. Recently, it has also become more practical and cost effective to use SNP-based tools for parentage verification. Some cattle breed associations, such as the US Jersey Association have begun to solely use SNPs for parentage verification. However, most breeds are just beginning the transition from MS to SNP markers. Traditionally, when a livestock industry transitions to a new technology for parentage verification, the additional cost of re-genotyping the transition generation(s) with the newer technology is absorbed by the producer or breed association. In an effort to reduce the cost of SNP technology adoption across cattle breeds, we initially developed a method to impute MS alleles from dense SNP genotypes (McClure et al., 2012). Our initial report in 4 dairy breeds (Holstein, Brown Swiss, Jersey, and Guernsey) found that 17% of the SNP-MS haplotypes were preserved across 2–4 of the studied breeds, suggesting that while many haplotypes are breed specific, some are present in phylogenetically distant breeds, possibly because they are identical by descent (IBD) from the common breed ancestor.

The objective of this study was to develop a SNP-MS haplotype reference panel set that could be used globally across the majority of commercial *Bos taurus* breeds and the major *B. indicus* breeds. An additional objective was to provide a data set and workflow so that any lab or service provider could implement our results for the benefit of the world-wide cattle community.

## Materials and methods

### Genotypes

Twenty-five groups, representing government, academic, and DNA service providers from the North American, South American, European, and Australian continents, including the International Bovine HapMap Project (International Bovine Hapmap Consortium, 2006) provided MS and partial Illumina BovineHD (Illumina Inc., 2010) (Illumina Inc., San Diego, CA, USA) genotypes on 16,564 animals representing 51 breeds plus 135 *B. taurus* crossbred animals (Table 1). All animals that were registered with their respective breed associations have accurate pedigree information which was available to this project. The provided genotypes were for SNP located within 500 kb (*N* = 3732) of 12 MS markers (*BM1818, BM1824, BM2113, ETH3, ETH10, ETH225, INRA023, SPS115, TGLA53, TGLA122, TGLA126, TGLA227*). These 12 MS loci comprise the International Society of Animal Genetics' (ISAG) recommended bovine parentage markers (http://www.isag.us/Docs/CattleMMPTest_CT.pdf) for inclusion in test panels used by service laboratories. All SNP data were captured and output in Illumina AB format. Genotypes for the ISAG-sanctioned MS bovine panel on the individuals and/or their parents were obtained from > 30 breed associations or their corresponding authorized data repositories. These MS genotypes were generated by multiple labs including GeneSeek (Lincoln, NE), MetaMorphix Inc. (Davis, CA), Maxxam (Mississauga, ON, Canada), UC Davis Veterinary Genetics Lab (Davis, CA), Zoetis (Kalamazoo, MI), Weatherbys DNA Laboratory (Kildare, Ireland), and Deoxi Biotecnologia (Araçatuba, São Paulo, Brazil), and LABOGENA (Jouy-en-Josas, France). Selected HapMap project individuals from less conventional or popular U.S. breeds were MS genotyped at UC-Davis Veterinary Genetics Lab, and Brahman individuals were MS genotyped by Zoetis according to ISAG genotyping standards.

**Table 1:** **Sample breed counts**.

**Breed**	**Count**			**Percent**
	**Reference**	**Validation**	**GGP-val**	**Total reference (%)**
Abondance	165	7	–	2.04
Angus	359	288	16	4.44
Ankole-Watusi	–	–	15	0.00
Aubrac	234	5	–	2.90
Ayshire	71	510	–	0.88
Bazadaise	53	27	–	0.66
Beefmaster	17	17	–	0.21
Belgian Blue	169	39	12	2.09
Belmont Red	–	40	–	0.00
Blonde D'Aquitaine	201	24	–	2.49
*Bos taurus* crossbred	29	106	–	0.36
Brahman	358	31	–	4.43
Brangus	8	–	–	0.10
Braunvieh	16	1	–	0.20
Bretonne Pie Noire	16	11	–	0.20
Brown Swiss	33	75	–	0.41
Brune Des Alpes	109	–	–	1.35
Charolais	1092	340	14	13.52
Chiangus	–	19	–	0.00
Devon	–	–	16	0.00
Dexter	–	–	15	0.00
Friesian	35	140	–	0.43
Gasconne	142	–	–	1.76
Gelbvieh	24	16	–	0.30
Gir	125	114	–	1.55
Guernsey	18	94	–	0.22
Hereford	251	589	–	3.11
Holstein	528	2103	5	6.54
Jersey	48	48	–	0.59
Kerry	–	1	–	0.00
Lagunair	–	5	–	0.00
Limousin	1599	557	–	19.80
Maine-Anjou	–	19	16	0.00
Montbeliarde	251	6	–	3.11
Murray Grey	–	22	–	0.00
Ndama	–	24	–	0.00
Nelore	124	1739	–	1.54
Normande	243	13	–	3.01
Norwegian Red	–	17	–	0.00
Parthenaise	218	73	–	2.70
Pie Rouge Des Plaines	116	44	–	1.44
Piedmontese	24	–	–	0.30
Red Angus	46	9	–	0.57
Romagnola	–	24	–	0.00
Rouge Flamande	41	–	–	0.51
Salers	234	24	–	2.90
Santa Gertrudis	–	97	–	0.00
Sheko	–	18	–	0.00
Shorthorn	17	170	–	0.21
Simmental	521	217	–	6.45
Swedish Red	2	3	–	0.02
Tarentaise	155	12	–	1.92
Texas Longhorn	–	–	13	0.00
Tropical Composite	336		–	4.16
Vosgienne	49	4	–	0.61
Unknown taurine	–	880	–	0.00
Total	8077	8622	122	100.00

From these MS and SNP genotypes, two populations were generated (Table 1). The reference population contained 8077 individuals from 39 breeds as well as 29 *B. taurus* crossbred animals with both MS and SNP genotypes. Seven to 12 (average of 9) MS genotype records were provided for each animal in the reference population, resulting in each MS having 2403–8031 genotyped individuals in this group (Table 2). The validation population was based on animals with only SNP data and contained 8622 animals representing 45 breeds and 106 *B. taurus* crossbred animals. MS genotypes on 1301 of the validation animals' parents, mainly sires, were also available for the evaluation of imputation accuracy. Only 89 validation animals had a parent present in the reference population. Both populations contained *B. taurus* and *B. indicus* purebreds and composite animals. BEAGLE (Browning and Browning, 2007) was used to impute the <2% of missing SNP genotypes in the reference and validation population. This step was considered robust based on previous reports where SNP genotypes were imputed with >95% accuracy with only a few hundred reference animals (Pausch et al., 2013) and with 98–99% accuracy in multi-breed reference populations (Larmer et al., 2010).

**Table 2:** **Microsatellite (MS) imputation haplotype information**.

										**Breed count per haplotype**^**i**^			
							**Haplotypes**			**100% MS**		**>90% MS**	
**MS**	**Chr**	**Alleles**^**a**^	**BT**^**b**^	**BI**^**c**^	**Haplotype**^**d**^	**Size**^**e**^	**Unique**^**f**^	**Tallied**^**g**^ **(%)**	**Population**^**h**^ **(%)**	**Ave**	**Max**	**Ave**	**Max**
*BM1824*	1	10	7035	963	70	264,604	551	50.8	91.9	2.42	19	7.53	27
*BM2113*	2	12	7069	962	50	143,822	1375	42.5	87.4	2.13	12	4.24	17
*INRA023*	3	15	7006	855	110	401,960	1158	38.9	87.7	1.78	11	4.18	21
*ETH10*	5	11	6875	962	80	377,892	582	46.7	92.6	2.34	14	6.04	27
*ETH225*	9	12	7055	960	110	429,755	1569	35.2	82.6	1.74	10	3.62	22
*SPS115*	15	13	6971	965	40	95,748	461	46.6	90.9	2.99	23	6.43	36
*TGLA53*	16	21	2902	302	80	299,752	403	26.8	57.1	1.80	10	3.13	16
*TGLA227*	18	18	7053	966	40	140,730	1071	39.6	85.6	1.94	18	5.40	18
*ETH3*	19	14	3850	101	80	267,330	491	30.1	67.7	1.46	6	3.15	10
*TGLA126*	20	12	6808	943	90	263,494	619	41.5	79.5	2.22	14	7.51	35
*TGLA122*	21	26	7056	966	50	145,453	605	42.4	86.5	2.31	17	5.98	33
*BM1818*	23	11	1463	940	80	269,309	525	42.1	88.6	4.52	12	3.43	11
Ave		15	5929	824	73	258,321	784	40	83	2.30	14	5.05	23

a Count of unique MS alleles observed in reference population.

b Count of Bos taurus reference animals with genotype for given MS.

c Count of Bos indicus reference animals with genotype for given MS.

d Number of SNPs included in haplotype centered on MS.

e Ba se pair size of the SNP haplotype.

f Number of unique SNP haplotypes in the reference population.

g Percent of haplotypes in the reference population that met the haplotype identification selection criteria.

h Percent of total BT reference population haplotypes that were tallied.

i Average number of breeds in the reference population having a MS-SNP haplotype where the SNP haplotype is linked to 1 MS 100% of the time or > 1 MS and matches 1 MS allele > 90% of the time.

A separate validation population (GGP-val) comprising of 122 animals from 9 breeds (Angus, Ankole-Watusi, Belgian Blue, Charolais, Devon, Dexter, Holstein, Maine-Anjou, and Texas Longhorn) was assembled to test MS imputation from the GGP-LD (GeneSeek Genomic Profiler Low Density) Beadchip (Neogen Corporation, 2012). While the GGP-LD contains ~80% of the original MS imputation SNP reported in McClure et al. (2012) these SNP genotypes were not imputed to the higher SNP density available in the reference population. These animals were also genotyped for the 12 MS at UC-Davis Veterinary Genetics lab.

### Haplotype estimation

BEAGLE input files for the reference population were created for each MS marker and flanking SNP within 500 kb. Animals were filtered on their MS genotypes so that for each MS the BEAGLE file contained only individuals with a MS genotype, thus 12 files were generated ranging from 2403 to 8031 animals (Table 2). All reference individuals were phased together using BEAGLE with 100 iterations. Williams et al., 2012 observed that phasing human ethnic groups together instead of separately resulted in increased phasing accuracy, as long as a single cohort did not dominate the dataset (>80% of the total population). Our reference population was fairly evenly distributed (Table 1) and each breed represented an average of 2.5% of the total population with only 2 breeds representing over 10% (Charolais at 13.5% and Limousin at 19.8%).

SNP haplotypes for MS imputation were identified using a similar process as reported in McClure et al. (2012). Optimal haplotype size for MS imputation was determined by analysing phased haplotypes, centered on the MS, using sliding windows that increased in size (10–20 flanking SNP increments). The number of unique reference population haplotypes that were linked to 1 MS allele 100% of the time and the number of haplotypes that were linked to >1 MS alleles but matched 1 MS allele ≥90% of the time were tallied. The optimal haplotype size was determined when either of the following criteria was met:

1. The maximum number of unique haplotypes appearing ≥4 times and linked to only 1 MS allele 100% of the time or linked to 1 MS allele ≥905 of the time across all breeds was obtained.
2. Increasing the haplotype size by 10 SNP resulted in ≤ 1% increase in the total number of tallied haplotypes.

### Imputation reference population creation

Two MS-SNP haplotype imputation reference populations were created from the full reference population using the optimal SNP haplotype size for each MS (Table 2). The *B. taurus* reference (BT-ref) population contained BT and BT crossbred animals with MS and SNP genotypes. The *B. taurus* + *B. indicus* imputation reference (BT + BI-ref) population contained BT, BT crossbred, and BI animals with MS genotypes and SNP genotypes. Each imputation reference population was then phased independently in BEAGLE as before.

### Microsatellite imputation

Two validation subpopulations, BT-val and BT + BI-val, were created from the validation population in the same manner as the imputation reference populations. Imputation was performed using either the 880 minimum SNP (min) panel (Table S1) from the optimal haplotype sizes identified above or all 3732 SNP within 500 kb of a MS marker (1 Mb). MS were imputed in BEAGLE using 11 different strategies:

1. BT-val, BT-ref, min, 20
2. BT-val, BT-ref, min, 100
3. BT-val, BT-ref, 1 Mb, 20
4. BT-val, BT + BI-ref, min, 20
5. BT-val, BT + BI-ref, min, 100
6. BT + BI-val, BT + BI-ref, min, 20
7. BT + BI-val, BT + BI-ref, min, 100
8. GGP-val, BT-ref, min, 20
9. GGP-val, BT-ref, min, 100
10. GGP-val, BT + BI-ref, min, 20
11. GGP-val, BT + BI-ref, min, 100

where the first, second, third and fourth term represent: validation population, reference population, SNP panel used, number of BEAGLE iterations.

### Mendelian inheritance conflicts of microsatellite alleles

For the 1301 validation population animals with submitted parental MS genotypes submitted, the animal's BEAGLE-imputed MS alleles were checked for Mendelian inheritance consistency against the MS genotype of its parents. Mendelian inheritance verification was also evaluated for 3457 reference population animals that had individual and parental MS genotypes submitted by the breed associations. An ANOVA was performed to determine statistical differences between the Mendelian consistencies of BT-val imputed MS and BT-ref reported MS genotypes, and between the different MS imputation parameter combinations. For the 122 GGP-val genotyped animals the concordance between their imputed and reported MS genotypes was determined. Both imputed MS alleles had to match the reported MS alleles to be considered concordant.

## Results

### MS haplotype imputation

**Figure 1:**
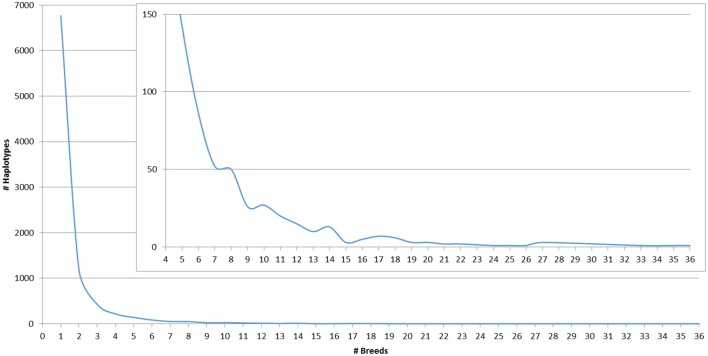
**Count of MS-SNP haplotypes present in at least one breed across the whole reference population**.

The number of SNP used for haplotype imputation for each MS ranged from 40 to 110 (average 73), with 83.16% of the reference population haplotypes being linked to only 1 MS allele 100% of the time or 1 MS allele ≥ 90% of the time across all breeds (Table S2). Less than 6% of the SNP haplotypes were associated with >1 MS allele and when this occurred, the other MS alleles were often within 2 bp of the most commonly associated allele (Table S3). These associations are potentially caused by a combination of rare haplotypes and MS genotyping errors, insertions and deletions within the amplified MS region that caused a rounding up or down of the called MS allele fragment size, or SNP haplotypes present in multiple breeds that are associated with multiple MS alleles in each breed due to recombination. On average, a haplotype that was associated with only 1 MS allele 100% of the time was present in 2.3 breeds with some such haplotypes being common across up to 23 breeds. For haplotypes that were associated with >1 MS allele, the most common MS allele was present in an average of ~7 breeds with a maximum of 36 breeds (Table 2). The distribution of MS-SNP haplotypes present in ≥1 breed across the whole reference population is shown in Figure 1. The large number of MS-SNP haplotypes observed only once or twice within the reference population are considered rare MS-SNP haplotypes (Table S3). While the majority of the MS-SNP haplotypes, 74.5%, were bred specific, the occurrence of 25.5% of the MS-SNP haplotypes being observed 2–36 breeds indicates that MS haplotype data from one breed can be informative for the imputation of MS alleles in other breeds.

### Imputation accuracies

The concordance between imputed and reported MS for the GGP-val animals averaged 72.05% in the *B. taurus* breeds when either the BT or BT × BI reference populations were used. MS concordance in the breeds with *indicine* ancestry such as Texas Longhorn and the Ankole-Watusi (Reist-Marti et al., 2003; McTavish et al., 2013) was greater when the BT × BI ref was used (concordance = 54.42% and 55.00%, respectively) compared to when only the BT-ref was used (concordance = 43.27% and 30.28%, respectively) (Table 3).

**Table 3:** **Microsatellite genotype concordance accuracies for animals with microsatellite alleles imputed from GGP-LD SNP**.

**Breed**	**Reference**^**a**^	**Iterations**^**b**^	**Ave (%)**	**Max (%)**	**Min (%)**
Angus	BT	20	80.73	100.00	58.33
		100	80.73	100.00	58.33
	BT + BI	20	80.73	100.00	58.33
		100	80.73	100.00	58.33
Belgian Blue	BT	20	72.92	91.67	50.00
		100	73.61	91.67	50.00
	BT + BI	20	73.61	91.67	58.33
		100	72.92	91.67	58.33
Charolais	BT	20	73.81	91.67	58.33
		100	75.00	91.67	58.33
	BT + BI	20	76.79	100.00	58.33
		100	76.19	91.67	58.33
Devon	BT	20	65.63	83.33	41.67
		100	66.67	83.33	41.67
	BT + BI	20	68.23	83.33	50.00
		100	69.79	83.33	50.00
Dexter	BT	20	61.11	83.33	41.67
		100	60.56	83.33	41.67
	BT + BI	20	60.00	83.33	41.67
		100	58.89	83.33	41.67
Holstein	BT	20	81.67	100.00	41.67
		100	81.67	100.00	41.67
	BT + BI	20	78.33	100.00	25.00
		100	78.33	100.00	25.00
Maine-Anjou	BT	20	66.15	91.67	41.67
		100	66.15	91.67	33.33
	BT + BI	20	68.75	91.67	41.67
		100	67.71	91.67	41.67
Texas Longhorn	BT	20	43.59	66.67	25.00
		100	42.95	66.67	25.00
	BT + BI	20	53.85	75.00	33.33
		100	53.85	75.00	33.33
Ankole-Watusi	BT	20	32.22	58.33	8.33
		100	28.33	41.67	8.33
	BT + BI	20	57.22	91.67	41.67
		100	52.78	83.33	33.33

The min SNP set was used for GGP imputations.

a BT, Bos taurus breeds; BT + BI, Bos taurus and Bos indicus breeds.

b Number of BEAGLE iterations.

While the parameters used for MS imputation: reference population, SNP haplotype size, or number of imputation cycles had no statistical effect (*P* > 0.98) on the Mendelian inheritance conflicts of the imputed MS (Table 4), the average computing time required for the different parameters combinations differed greatly, ranging from under 1 min to over 3 h per MS (Table 5). A statistical difference (*P* ≤ 0.04) existed between the Mendelian inheritance consistencies of BT-val imputed MS (average 95.3%) and BT-ref reported MS (average 97.8%) (Table 4). On average, 68.09% of the 1291 BT-val animals with imputed MS had no Mendelian inheritance conflicts with their parents' MS genotype, 22.83% had only 1 conflict, 4.95% had only 2 conflicts and 4.13% had >2 conflicts. In comparison, the 3457 reference animals with parental MS data had 85.25% with no conflicts, 10.65% with 1 conflict, 2.34% with 2 conflicts, and 1.76% with >2 conflicts (Table 6). There was variability in the average Mendelian inheritance accuracy of imputed MS among breed and MS in the validation population with an average breed accuracy of 94% across all imputation strategies (Table 6).

**Table 4:** **Mendelian inheritance accuracy by microsatellite and imputation strategy**.

	**Validation population**										**Reference population**			
**Validation**^**a**^			**BT**	**BT**	**BT**	**BT**	**BT**	**BT + BI**	**BT + BI**					
**Reference**	**BT**	**BT + BI**	**BT**	**BT**	**BT**	**BT + BI**	**BT + BI**	**BT + BI**	**BT + BI**		**BT**	**BT + BI**	**BT**	**BT + BI**
**SNP**^**b**^			**min**	**min**	**1 Mb**	**min**	**min**	**min**	**min**					
**Iterations**^**c**^			**20**	**100**	**20**	**20**	**100**	**20**	**100**					
**Marker**	**count**^**d**^		**% acc**^**e**^							**Average (%)**	**Count**^**f**^		**% acc**^**g**^	
*BM1818*	777	786	95.50	95.88	96.65	96.91	**97.04**	96.95	96.69	96.52	552	859	98.91	98.84
*BM1824*	1283	1293	96.34	96.34	**97.35**	96.49	96.65	96.37	96.52	96.58	3129	3446	98.98	98.69
*BM2113*	1263	1273	95.80	95.57	92.32	96.28	96.28	**96.39**	**96.39**	95.57	3099	3416	98.52	98.59
*ETH10*	1226	1237	96.41	96.08	96.82	**96.98**	96.90	96.93	96.77	96.70	3037	3352	98.52	98.57
*ETH225*	1274	1285	95.84	96.15	96.70	96.86	**96.94**	96.26	95.95	96.39	3115	3432	98.81	98.63
*ETH3*	1113	1121	**96.86**	96.59	96.41	96.41	96.32	96.34	96.61	96.50	1913	1923	98.01	97.97
*INRA023*	1254	1263	97.13	**97.53**	97.37	96.81	96.97	96.83	96.91	97.08	3055	3322	98.13	98.07
*SPS115*	1270	1281	96.85	96.77	96.30	96.85	96.61	**96.96**	**96.96**	96.76	3083	3400	98.28	97.35
*TGLA122*	1281	1292	96.17	96.25	95.86	96.64	96.41	96.67	**96.98**	96.43	3127	3444	98.27	98.17
*TGLA126*	1269	1280	90.39	89.99	**95.04**	90.31	90.39	90.63	90.55	91.04	3094	3411	96.22	96.22
*TGLA227*	1267	1278	94.71	94.48	**95.19**	93.69	93.84	94.84	94.60	94.48	3093	3408	98.03	98.06
*TGLA53*	1082	1084	89.74	89.28	**93.62**	89.00	88.72	89.48	88.93	89.83	1676	1734	93.38	92.16
Average	1197	1206	95.14	95.08	95.80	95.27	95.26	95.39	95.32	95.32	2664	2929	97.84	97.61

a BT, Bos taurus breeds, BT + BI, Bos taurus and Bos indicus breeds.

b min, minimum SNP haplotype, 1 Mb, full SNP haplotype.

c Number of BEAGLE iterations.

d Count of validation animals with reported parent microsatellite allele.

e Average % accuracy between individual's imputed microsatellite allele and reported parental allele; bold, highest imputed microsatellite accuracy.

f Count of reference animals with MS alleles and with reported parent microsatellite allele.

g Average % BT accuracy between individual's reported microsatellite allele and reported parental allele.

**Table 5:** **BEAGLE running time for reference and validation populations**.

**Marker**	**Validation**^**a**^	**BT**	**BT**	**BT**	**BT**	**BT**	**BT + BI**	**BT + BI**	**GGP**	**GGP**	**GGP**	**GGP**	**−**^**d**^	
	**Reference**	**BT**	**BT**	**BT**	**BT + BI**	**BT + BI**	**BT + BI**	**BT + BI**	**BT**	**BT**	**BT + BI**	**BT + BI**	**BT + BI**	**BT**
	**SNP**^**b**^	**min**	**min**	**1 Mb**	**min**	**min**	**min**	**min**	**min**	**min**	**min**	**min**	**1 Mb**	**1 Mb**
	**Iterations**^**c**^	**20**	**100**	**20**	**20**	**100**	**20**	**100**	**20**	**100**	**20**	**100**	**100**	**100**
	**chr**	**Time (hour:minute:second)**												
*BM1824*	1	0:01:04	0:03:54	0:23:04	0:01:15	0:04:45	0:03:22	0:15:05	0:00:15	0:01:04	0:00:17	0:01:16	0:38:03	0:29:02
*BM2113*	2	0:02:49	0:09:58	4:57:47	0:03:01	0:10:23	0:07:03	0:33:25	0:00:29	0:02:10	0:00:33	0:02:30	2:07:16	1:37:04
*INRA023*	3	0:03:08	0:10:47	0:21:30	0:03:49	0:13:09	0:19:04	1:24:35	0:01:11	0:05:20	0:01:27	0:06:55	0:45:42	0:33:10
*ETH10*	5	0:01:24	0:05:14	0:05:27	0:01:30	0:05:57	0:04:36	0:18:22	0:00:19	0:01:25	0:00:22	0:01:42	0:07:12	0:06:15
*ETH225*	9	0:04:22	0:16:37	0:20:07	0:04:57	0:18:20	0:17:57	1:16:59	0:01:13	0:05:34	0:02:01	0:09:46	0:29:53	0:23:06
*SPS115*	15	0:01:22	0:05:00	5:03:09	0:01:06	0:04:02	0:03:27	0:15:57	0:00:16	0:01:12	0:00:22	0:01:43	1:50:45	1:29:21
*TGLA53*	16	0:02:13	0:06:55	0:13:49	0:02:21	0:07:32	0:07:36	0:27:19	0:00:09	0:00:40	0:00:11	0:00:47	0:08:31	0:06:59
*TGLA227*	18	0:01:51	0:06:46	3:17:29	0:02:04	0:07:07	0:07:24	0:27:45	0:00:13	0:00:59	0:00:16	0:01:07	1:24:19	1:04:29
*ETH3*	19	0:01:31	0:05:04	0:19:39	0:01:38	0:05:33	0:04:45	0:20:29	0:00:21	0:01:41	0:00:25	0:01:55	0:14:01	0:12:49
*TGLA126*	20	0:01:38	0:06:20	0:46:27	0:01:52	0:07:18	0:05:17	0:22:56	0:00:18	0:01:26	0:00:23	0:01:49	0:48:23	0:35:20
*TGLA122*	21	0:01:15	0:04:06	0:51:59	0:01:17	0:03:56	0:03:51	0:11:58	0:00:11	0:00:45	0:00:13	0:00:49	0:40:33	0:31:13
*BM1818*	23	0:00:52	0:03:01	0:22:31	0:01:00	0:03:26	0:02:04	0:07:11	0:00:03	0:00:10	0:00:04	0:00:14	0:09:05	0:05:08
	Average	0:01:57	0:06:59	1:25:15	0:02:09	0:07:37	0:07:12	0:30:10	0:00:25	0:01:52	0:00:33	0:02:33	0:46:59	0:36:10

a BT, Bos taurus breeds; BT + BI, Bos taurus and Bos indicus breeds.

b min, minimum SNP haplotype, 1 Mb, full SNP haplotype.

c Number of BEAGLE iterations.

d CPU running time for reference population.

**Table 6:** **Average Mendelian inheritance accuracy by microsatellite and breed across all imputation strategies**.

**Breed**	**Animal count**	**BM1818**	**BM1824**	**BM2113**	**ETH10**	**ETH225**	**ETH3 )**	**INRA023**	**SPS115**	**TGLA122**	**TGLA126**	**TGLA227**	**TGLA53**	**Overall**
		**(%)**	**(%)**	**(%)**	**(%)**	**(%)**	**(%)**	**(%)**	**(%)**	**(%)**	**(%)**	**(%)**	**(%)**	**(%)**
Angus	58	100.00	97.78	99.74	96.43	96.80	97.67	98.28	98.15	98.28	98.25	91.43	87.04	95.44
Bos taurus crossbred	38	99.21	96.88	95.67	73.56	94.64	–	99.31	96.88	98.81	96.78	99.40	–	95.11
Beefmaster	4	–	100.00	100.00	100.00	100.00	100.00	–	100.00	100.00	100.00	100.00	100.00	100.00
Belgian Blue	3	–	100.00	71.43	100.00	100.00	100.00	100.00	100.00	100.00	100.00	100.00	100.00	97.40
Belmont Red	6	73.81	90.48	83.33	83.33	78.57	–	100.00	95.24	100.00	90.48	100.00	–	89.52
Brahman	4	100.00	100.00	100.00	100.00	50.00	–	100.00	75.00	100.00	100.00	100.00	–	89.29
Braunvieh	1	–	100.00	100.00	100.00	100.00	100.00	–	100.00	100.00	100.00	14.29	100.00	91.43
Charolais	112	100.00	95.79	97.19	100.00	98.47	96.78	97.58	100.00	92.35	100.00	97.92	96.49	97.71
Chiangus	1	–	100.00	100.00	85.71	100.00	100.00	100.00	100.00	100.00	100.00	100.00	100.00	98.70
Freisan	16	80.95	95.54	98.21	83.04	100.00	100.00	99.11	92.86	93.75	92.86	100.00	75.82	92.68
Gelbvieh	1	100.00	14.29	100.00	100.00	100.00	100.00	100.00	100.00	100.00	100.00	100.00	14.29	85.71
Hereford	473	97.85	95.65	95.68	97.80	96.71	97.61	96.32	95.22	96.53	83.75	95.86	90.76	94.98
Holstein	61	85.71	99.77	99.05	99.30	98.13	99.77	100.00	100.00	98.36	98.59	87.76	96.92	96.95
Jersey	12	100.00	95.24	100.00	51.19	81.43	100.00	100.00	100.00	100.00	92.21	97.40	98.57	93.00
Limousin	106	94.56	98.64	94.74	97.64	99.46	100.00	98.02	99.45	95.33	98.64	95.78	93.09	97.11
Nelore	14	100.00	100.00	100.00	100.00	85.71	100.00	100.00	100.00	100.00	100.00	100.00	–	98.70
Red Angus	2	–	100.00	100.00	100.00	100.00	100.00	100.00	100.00	100.00	92.86	100.00	14.29	91.56
Simmental	74	94.49	98.65	97.02	99.21	99.32	100.00	97.01	94.03	95.74	78.91	97.06	79.29	94.23
Unknown taurine	326	93.99	97.14	93.37	97.78	93.90	91.49	96.57	97.36	96.93	95.74	91.22	85.23	94.23
Overall	1312	94.33	93.46	96.08	92.89	93.32	98.96	98.95	97.06	98.21	95.74	93.06	82.12	94.41

For the 25 BT-val animals with a parent in the reference population and a MS conflict, if the matching SNP haplotypes are taken into consideration, 17 have 100% parent verification. Only 7 animals had 1 haplotype conflict (i.e., 1 MS conflict) and one animal had 2 haplotype conflicts. Taking the matching SNP haplotypes into consideration means that for the 89 validation animals with a parent in the reference population, 91% have no MS or SNP haplotype conflicts, 98.88% have ≤1 conflict and 100% have ≤2 conflicts. These conflict statistics are higher than the MS parent verification statistics for the BT- ref animals in Table 7.

**Table 7:** **Average Mendelian inheritance accuracy for different imputation methods**.

**Population**	**Subset**^**f**^		**Validation population**^**a**^**(%)**							**Ref population**^**b**^**(%)**	
		**Validation**^**c**^	**BT**	**BT**	**BT**	**BT**	**BT**	**BT + BI**	**BT + BI**		
		**Reference**	**BT**	**BT**	**BT**	**BT + BI**	**BT + BI**	**BT + BI**	**BT + BI**	**BT + BI**	**BT**
		**SNP**^**d**^	**min**	**min**	**1 Mb**	**min**	**min**	**min**	**min**	**(N = 3457)**	**(N = 3140)**
		**Iterations**^**e**^	**20**	**100**	**20**	**20**	**100**	**20**	**100**		
		**Conflicts**^**g**^									
Bt	All (*N* = 1291)	0	66.54	67.39	72.42	67.39	66.46	68.32	68.16	85.25	86.66
		≤1	90.55	90.40	91.25	91.17	91.32	91.09	90.70	95.89	96.31
		≤2	95.58	95.20	96.05	96.05	96.05	96.05	96.13	98.24	98.31
	Sire/dam not ref^h^ (*N* = 1202)	0	66.56	67.55	72.21	67.55	66.56	68.55	68.64		
		≤1	90.35	90.35	90.93	90.93	91.10	90.77	90.52		
		≤2	95.59	95.17	96.09	96.09	96.09	96.01	96.09		
	Not reference breed^i^ (*N* = 368)	0	59.24	58.97	71.47	61.14	59.78	61.96	62.23		
		≤1	85.05	84.51	88.32	87.77	87.77	88.04	87.23		
		≤2	93.75	91.58	94.84	94.84	94.84	95.38	95.11		
BT × BI	All (*N* = 11)	0						81.82	81.82		
		≤1						90.91	90.91		
		≤2						90.91	100.00		

a Average accuracy for the validation populations using imputed microsatellite alleles and their parents' reported alleles.

b Average accuracy for the reference population and their parents using reported microsatellite alleles.

c BT, Bos taurus breeds; BT + BI, Bos taurus and Bos indicus breeds.

d min, minimum SNP haplotype; 1 Mb, full SNP haplotype.

e Number of BEAGLE iterations.

f Subset of individuals in the validation population whose parents have reported microsatellite genotypes.

g Total number of imputed microsatellite alleles with Mendelian inheritance conflicts.

h The animal's parents were not part of the reference population.

i The animal's breed is not represented in the reference population.

## Discussion

Imputation accuracy did not statistically differ among the combinations of imputation parameters, although the CPU time required for imputation was much greater when all SNPs flanking 500 kb each side of the MS were included in the imputation process compared to when the most parsimonious number of flanking SNPs were used (Tables 4, 5, 7). While the imputed MS alleles showed greater Mendelian inheritance conflicts than the reported MS alleles did, this was expected as previous research has documented that MS marker genotypes themselves have a 1–5% error rate and only 85% of the reference animals had no parentage MS conflicts.

An analysis of the SNP haplotypes for the 25 BT-val animals with Mendelian inheritance conflicts and with sires in the BT-ref population indicated that many of their SNP haplotypes were not in conflict (Table S4). In these cases, the sire haplotype may have harbored a mis-scored MS allele. For instance, Table S4 (Tab *TGLA126*) shows the *TGLA126* SNP haplotypes for Simmental-679 and its sire (Simmental-334), the imputed MS genotypes for Simmental-679 (*123/115*) were in conflict with its sire's reported genotype (*117/117*), even though both animals share a common haplotype. When the shared SNP haplotype was examined in Table S3 (Tab chr20-*TGLA126*, column UP) the most common MS allele observed for this haplotype is *123*. The haplotype was associated with the *123* allele 937 times (99.68%) across 17 breeds and the *117* allele only once (0.11%). While it is possible that the sire's reported MS genotype is correct, it appears to be more likely that the sire's genotype was incorrectly scored. This 0.11% error rate is within reported MS error rates found in literature (Baruch and Weller, 2008). Of note, the other *TGLA126* SNP haplotype for this sire was associated with the *117* allele 301 times (88.79%) across 11 breeds (Table S3, tab chr20-TGLA126, column VI). It is possible that when this animal was genotyped the *123* allele failed to PCR amplify, amplified too weakly to be called, or simply failed to be called, such that the animal was genotyped as *117* homozygote, instead of *117/123*.

### Recommendations

The optimized SNP haplotypes reported here and the reference population data represent a robust standard data set that can be used to impute MS at high accuracy (Table 4, average 95%) for the loci within the ISAG recommended bovine parentage MS panel. This standard can be used in breeds that are not represented in the reference panel with only a small reduction in accuracy (Table 7).

For the research reported here to be implemented by the industry we suggest the following work flow:

1. Genotype animals with a SNP assay that contains our reported min SNP set (Table S1) and parentage SNP (Heaton et al., 2002; Werner et al., 2004) panels. These include the BovineHD, GeneSeek Genomic Profiler Bovine HD (GGP-HD), Super-GGP (Neogen Corporation, 2013), or the International Dairy and Beef (IDB) assays (Berry et al., 2013).
2. If the animal's parents have parentage SNP genotypes then parentage verify with SNP data.
3. If parents have no parentage SNP data then either:
  - Impute the animal's MS genotype via BEAGLE using the min SNP set and BT-ref as the reference population. If the animal is a *B. indicus* purebred or crossbred then use BT × BI as the reference population for haplotype reconstruction.
  - Phase the SNP with BEAGLE, fastphase (Scheet and Stephens, 2006), findhap (Vanraden, 2011), HAPI_UR (Williams et al., 2012), or other appropriate program. Then match the haplotype with the appropriate MS tab in Table S3 and return the most common MS allele to impute the animal's MS genotype.
4. Use the imputed MS genotypes for parentage verification.
5. If parentage verification fails, then genotype the animal with MS panel.
  - If the actual and imputed MS genotypes match, then consider retesting the parent with MS to correct the genotype error.
  - If the actual and imputed MS genotypes do not match, then phase the animal's SNPs and MS genotypes and add this animal to the reference population.
6. Generate an updated reference haplotype population by adding any new animal with actual MS and SNP genotype data to the reference population dataset and rephrase all of the SNP and MS genotypes.
7. Use the updated reference population at Step 3.

By MS genotyping the animal if a discrepancy occurs the process described above will self-correct for MS genotyping errors and capture rare MS-SNP haplotypes Generation of new reference panels (Step 6 above) will help: A) increase the imputation accuracy, and B) to identify rare or breed specific MS-SNP haplotypes. This process will also speed up the adoption of the accurate 101 SNP panel (Heaton et al., 2002) or derivative for parentage verification over the current MS panel.

For individuals that solely wish to parentally verify an individual and transition between MS and SNP genetic markers it currently would be most cost effective for one to genotype the animal with the ISAG MS panel ($15-€20) and a 116 SNP panel ($15) than to use a Super-GGP, GGP-HD, BovineHD, or IDB beadchip (€30-$185) (Jeremy Walker, GeneSeek, and John Flynn, Weatherbys, Pers. Commun., 22/07/2013). For those wishing to obtain genomic breeding values, select genetic disease status, and parentage SNP and MS genotypes on an animal than the listed beadchips and MS imputation do represent an economically viable option as one will not have to incur an additional cost to obtain MS genotypes.

As part of this international collaborative effort, the phased reference population data (BT-ref and BT + BI-ref) and marker (1 Mb and Min) BEAGLE files are available (Supplementary Data Sheets 1–3) to facilitate MS imputation in DNA service laboratories world-wide. Our results demonstrate the power of continued data sharing of MS and SNP genotypes from the BovineSNP, GGP-HD, Super-GGP, or IDB panels for the SNP genotypes within 500 kb of each MS to increase imputation accuracy. The haplotypes reported for these reference populations can be applied to accurately impute MS alleles with high accuracy on animals that have been genotyped for the flanking SNP, regardless of breed.

### Conflict of interest statement

The authors declare that the research was conducted in the absence of any commercial or financial relationships that could be construed as a potential conflict of interest.
